# Metallic Scaffolds for Bone Regeneration

**DOI:** 10.3390/ma2030790

**Published:** 2009-07-23

**Authors:** Kelly Alvarez, Hideo Nakajima

**Affiliations:** 1 Center for Geo-Environmental Science, Faculty of Engineering and Resource Science, Akita University, 1-1 Tegata Gakuen-machi, Akita 010-8502, Japan; E-Mail: kelly@gipc.akita-u.ac.jp; 2 The Institute of Scientific and Industrial Research, Osaka University, Ibaraki, Osaka 567-0047, Japan

**Keywords:** metallic bone scaffolds, biocompatible metals, load-bearing porous structures, 3-D metallic constructs, bone tissue engineering

## Abstract

Bone tissue engineering is an emerging interdisciplinary field in Science, combining expertise in medicine, material science and biomechanics. Hard tissue engineering research is focused mainly in two areas, osteo and dental clinical applications. There is a lot of exciting research being performed worldwide in developing novel scaffolds for tissue engineering. Although, nowadays the majority of the research effort is in the development of scaffolds for non-load bearing applications, primarily using soft natural or synthetic polymers or natural scaffolds for soft tissue engineering; metallic scaffolds aimed for hard tissue engineering have been also the subject of *in vitro* and *in vivo* research and industrial development. In this article, descriptions of the different manufacturing technologies available to fabricate metallic scaffolds and a compilation of the reported biocompatibility of the currently developed metallic scaffolds have been performed. Finally, we highlight the positive aspects and the remaining problems that will drive future research in metallic constructs aimed for the reconstruction and repair of bone.

## 1. Introduction

Human skeletal tissues have complex three-dimensional (3-D) geometries and highly organized internal architectures, which cannot be simply emulated by cells maintained in two-dimensions. Bone is a complex porous composite structure with specific characteristics such as viscoelasticity and anisotropy, both in morphology and mechanical properties [1]. The unique mechanical performance of natural bone is characterized by high toughness, high specific strength, and low stiffness. Porous scaffolds are central to hard tissue engineering strategies because they provide a 3-D framework for delivering reparative cells or regenerative factors in an organized manner to repair or regenerate damaged tissues. Since hard tissues are responsible for the body mechanical stability, materials aimed for repairing, substitution and/or restoration of hard tissues must possess strength, resistance to corrosion/degradation, have a good biocompatibility and exhibit good wear resistance.

The development of successful scaffolds for bone tissue engineering requires a concurrent engineering approach that combines different research fields. During the last three decades, researchers have tailored metallic scaffolds that are useful for a wide variety of medical and dental applications. Surface modification of already proved biocompatible metals is an essential requisite for the utilization to tissue engineering because the metal surface must be controlled to induce the adhesion and proliferation of cells and the adsorption of essential biomolecules.

In this literature review, we will summarize the progress and the state-of-the-art of the metallic scaffolds as well as the reported biocompatibility of each of these metallic structures that has been conceived to be used in specific reconstruction of small or large bone defects. The design of a hard tissue-engineered scaffold logically begins with an intensive characterization of the host tissue properties. The properties of bone and how these apply to the design of a synthetic scaffold are discussed below.

## 2. Bone Structure and Properties

**Figure 1:**
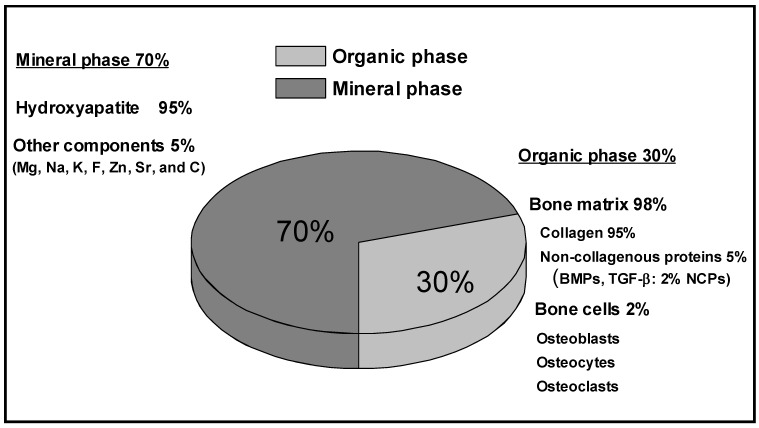
Chemical composition of bone tissue.

Bone is a natural composite material, which by weight contains about 45-60% minerals, 20-30% matrix, and 10-20% water. By including the water fraction in the organic phase, the composition of bone can be simply represented as shown in Figure 1. The matrix is the organic component, which is primarily composed of the protein Type I collagen [2]. Type I collagen is a triple helix that is highly aligned, yielding a very anisotropic structure. The non-collagenous proteins are composed of non-collagenous glycoproteins and bone specific proteoglycans, these proteins include osteocalcin, osteonectin, bone phosphoproteins, bone sialoproteins and small proteoglycans [2]. The non-collagenous proteins have different functions in the regulation of bone mineralization and cell-to-matrix binding interactions with structural proteins. Less than 1% of the non-collagenous proteins contain growth factors influencing the cells but also secreted by them [3]. The cellular component is made of osteoblasts (bone-forming cells), osteoclasts (bone-destroying cells), osteocytes (bone-maintaining cells, which are inactive osteoblasts trapped in the extracelullar matrix) and bone lining cells (inactive cells that are believed to be osteoblasts precursors) [4]. The mineral, inorganic component of bone is a calcium phosphate known as Hydroxyapatite (HA). Hydroxyapatite has a chemical structure represented by the formaula Ca_10_[PO_4_]_6_[OH]_2_ and is present in small crystallites form of approximately 2 × 2 × 40 nm^3^. These crystals undergo important changes in composition with age, thus their biologic functions depend on the amount and the age of the mineral crystals [5]. The inorganic matrix performs two essential functions as an ion reservoir and a structure giving the bone its stiffness and strength. In simple words, the organic matrix provides bone its flexibility and the inorganic material is predominantly responsible for the mechanical properties of bone [6,7].

The human skeleton can be categorized into two types of bone: the cortical bone and the trabecular bone. Although both bone types comprise the same composition, each one contains different proportions of the organic and inorganic materials, degree of porosity and organization. In addition, the combination of cortical and trabecular bone varies according the skeleton regions, which is dependent on the applied mechanical loading. Both, cortical and trabecular bones display time-dependent mechanical behavior, as well as damage susceptibility during cyclic loading [8,9].

Despite the multiple functions bone has in the body, its biomechanical role is the most compromised upon injury. Indeed, the other bones in the body can compensate for the injured bone’s metabolic function, but if a bone is broken or injured, it can no longer support the load it is meant for, and the body remains handicapped.

The mechanical properties of cortical bone have been well documented [10,11,12,13]. They can be measured via traditional testing techniques such as: uniaxial compressive or tensile testing, or three or four-point bending. Cortical bone exhibits a high degree of anisotropy and values of mechanical properties vary between animal species, bone location and testing conditions, age and disease. Testing conditions, for example, may vary between testing dry samples, testing wet samples at 37 °C and embedding them in an special resin or not.

Measuring properties of trabecular bone is far more complex than in the case of cortical bone as shown in Table 1. The complexity is due to the small dimensions of the individual trabeculae. When considered mechanically cortical and trabecular bone are not the same material. It is speculated that differences in moduli between cortical and trabecular bone are entirely due to the bone density. The range of cortical bone densities reported for the human proximal femur is 1.5-2 g/cm^3^ [14] and the range of apparent trabecular bone density in human proximal femora is 0.2-0.6 g/cm^3^ [15]. With either testing technique the mean trabecular Young's modulus is found to be significantly less than that of cortical bone. However, as can be seen in Table 1, some authors have found a value of elastic modulus of trabecular bone as high as those for cortical bone apparently because the test specimens were dried before the mechanical tests [12,16,18,19].

Mechanical properties of human bone depend dramatically on age; 3, 5, and 35-years old femoral specimens had a Young’s modulus of 7.0, 12.8 and 16.7 GPa, respectively [20]. Besides age, the nutritional state, physical activity (mechanical loading), bone related diseases, etc., will influence the properties of bone tissue. This fact establishes a major challenge in the design and fabrication of scaffolds aimed to repair specific sites in specific patients.

**Table 1:** Mechanical properties of human cortical and trabecular bone.

Cortical Bone	Shear Strength × 10^6^ N/m^2^	Strength × 10^6^ N/m^2^	Young’s Modulus range × 10^9^ N/m^2^
Compression test	-	219 ± 26 Longitudinal 153 ± 20 Transverse	14.1 – 27.6
Tensile test	-	172 ± 22 Longitudinal 52 ± 8 Transverse	7.1 – 24.5
Torsional test	65 ± 9	-	-
Ultrasonic method	-	-	22 – 24.5
**Trabecular Bone**	**Shear Strength × 10^6^ N/m^2^**	**Strength range** **× 10^6^ N/m^2^**	**Young’s Modulus** **range × 10^9^ N/m^2^**
Compression test	-	1.5 – 9.3	0.1 – 0.4
Tensile test	-	1.6 – 2.42	10.4 ± 3.5
Torsional test	6.35 ± 2	-	-
Ultrasonic method	-	-	14.8 ± 1.4

Compiled from references [10,11,12,13,17,19,21].

## 3. Bone Tissue Engineering

The goal of bone tissue engineering is to repair bone defects, which are difficult or even impossible to treat by conventional methods. This usually involves the use of 3-D bone graft substitutes to treat bone losses due to traumatic injury or revision surgery to augment the natural regenerative capacity of the body [22,23]. Bone tissue engineering employs a multidisciplinary approach, drawing on the principles of cell biology, molecular development biology, materials science and biomechanics, to aid in the repair of tissues damaged beyond the natural healing capacity of the bone. There are several approaches to bone engineering, ranging from inorganic bone fillers (in common clinical use) [24] to *in situ* bone induction by bone-inductive growth factors (in limited use) [25,26] to laboratory cultured bone cells and gene therapy (in experimental phase) [27,28,29]. All these methods, however, have two common requirements: a physical continuity across the damage site that has to be provided to guide the bone growth, and the avoiding of scar formation.

In general, three essential elements are needed to successfully engineer a biological tissue or organ:

1) Tissue forming cells (osteogenic cells) and/or signaling biomolecules
2) Biocompatible scaffolds conducive to normal cell functions, and
3) Quantitative measures of tissue’s regenerative outcome.

**Figure 2:**
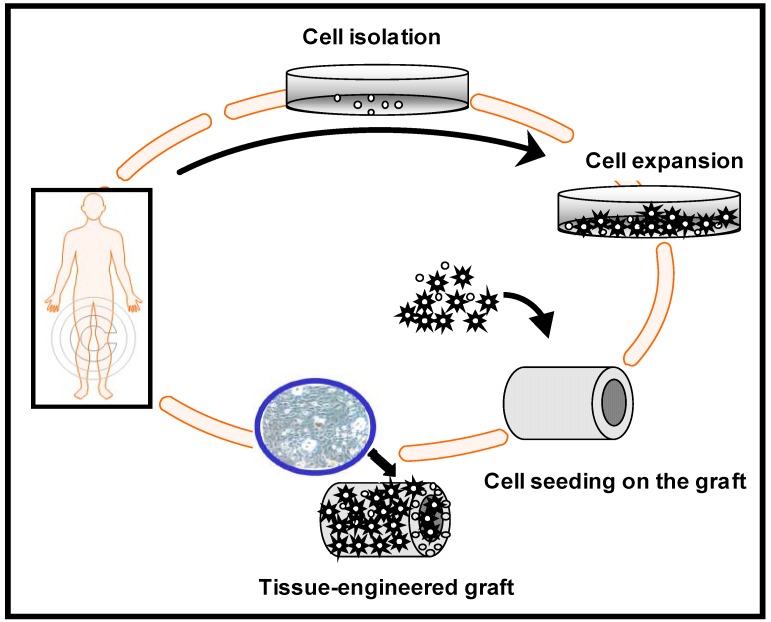
Tissue-engineered graft fabrication process.

An ideal strategy for the tissue engineering of bone is the harvesting of osteogenic cells from the patient, which are then expanded in culture and seeded on a scaffold or graft that act as a guide and stimulus for tissue ingrowth in 3-D (Figure 2). Ideally, the need to regenerate tissue can be forecasted in advance, and cells taken from the patient can be seeded onto a scaffold, grown *in vitro*, and then reimplanted back into the patient, resulting in a healing of the damaged tissue. In a tissue-engineered scaffold, mesenchymal stem cells (MSCs) are usually included to give rise to bone cells. These stem cells can be readily extracted from the bone marrow of adult mammals (including humans), and can be induced to differentiate into natural tissue. The scaffold material can be preseeded *in vitro* with osteogenic cells to promote bone formation. At the implant site these cell/scaffold constructs contribute to bone formation.

**Figure 3:**
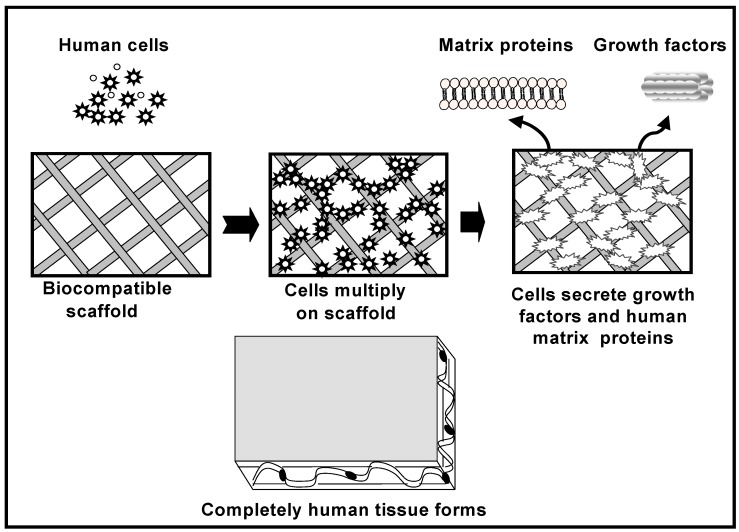
Cell-based tissue regeneration approach for the repair of bone defects.

The role of the scaffold is to act as a carrier that restricts the movement of these MSCs cells away from the implantation site and to provide support for new bone formation. Figure 3 schematically illustrates the cell-based strategy for tissue regeneration. The osteogenic cells lay down bone extracellular matrix in the surface of the scaffold as woven immature bone. Over time, a mature bone structure will form inside and in the exterior part of the scaffold allowing the regeneration of the tissue.

Growth factors such as basic fibroblasts growth factors (FGFs), platelet-derived growth factor (PDGF), insulin-like growth factor (IGF), epidermal growth factor (EPG), transforming growth factor-beta (TGF-β), bone morphogenetic proteins (BMPs), etc., also would be applied in the tissue engineered scaffolds to promote bone formation. When the scaffold material is loaded with specific bone-inductive growth factors, these exogenous growth factors are then released at the implantation site, where they can act upon locally resident cells as well as recruiting other more distant cells to form new bone tissue. Bone morphogenetic proteins are active bone-inducing factors that act on immature mesenchymal cells, including osteoblasts, resulting in osteogenesis [30]. To date, molecular cloning has isolated several types of BMPs, and recombinant BMP molecules have been synthesized [31]. BMPs 2, 4, 6, and 7 are generally considered to be the most osteoconductive of the bone morphogenetic proteins. BMP-2, specifically promotes undifferentiated mesenchymal cells into osteoblasts, leading to bone formation [32].

While these factors place special demands on all aspects of tissue engineering, scaffold design takes on a role of particular importance. We will discuss this topic separately in the following section.

Finally, postoperatively high quality image examinations are required to investigate the effectiveness of the implantation such as the position of the scaffold and evaluate the developing status of surrounding anatomic structures. For the clinical determination of the bone ingrowth inside the scaffold recently advances of the X-ray micro-computed tomography (μCT) imaging have shown sufficient resolution for the accurate identification of the bone ingrowth within the metallic porous structure. However, the complex process of bone remodeling inside a tissue-engineered construct, made up of scaffold material, host bone, mineralized bone and soft tissue, makes the partitioning of the tomogram into discrete phases non-trivial [33]. In the past, μCT was not suitable for metallic scaffolds as the metal heavily attenuates X-rays. The presence of metal resulted in dark and bright grainy artifacts, which obscure important details of the scan images [34]. However, improved algorithms for metal artifact reduction has been developed recently [35,36], and the combination of 2 mm thick aluminum filter and a 10 mm thick polymethylmethacrylate filter has been employed improving the signal-to-noise ratio in the images. By doing this it can be reduced the streak artifacts caused by the metallic material [37].

## 4. General Desirable Properties of the Bone Scaffolds

A scaffold for hard tissue reconstruction is a three dimensional construct, which is used as a support structure allowing the tissues/cells to adhere, proliferate and differentiate to form a healthy bone/tissue for restoring the functionality. In almost all the clinical cases, scaffolds for hard tissue repair in a load-bearing area are not temporary, but permanent. They most retain their shape, strength and biological integrity through the process of regeneration/repair of the damaged bone tissue. Bone replacement constructs for bone defects reconstructions would need to be biocompatible with surrounding tissue, radiolucent, easily shaped or molded to fit perfectly into the bone defect, non-allergic and non-carcinogenic, strong enough to endure trauma, stable over time, able to maintain its volume and osteoconductive (able to support bone growth and encourage the ingrowth of surrounding bone) [38,39,40,41,42].

Apart from the above-mentioned material requirements, the structural requirements expected for the possible candidate for bone scaffold are numerous, ranging from the maximum feasible porosity to the porous architecture itself. Pore size and interconnectivity are important in that they can affect how much cells can penetrate and grow into the scaffold and what quantity of materials and nutrients can be transported into and out of the scaffold. In other words, pore size distributions, porosity and the interconnectivity of the scaffold should be sufficient for cell seeding, cell migration, matrix deposition, vascularization and mass transport of nutrients from and to the cells. Physiologically, previous research has shown that the optimum pore size for promoting bone ingrowth is in the range of 100-500 μm [43,44,45]. However, the scientific community has not reached yet a consensus regarding the optimal pore size for bone ingrowth.

From a mechanical perspective, scaffold materials aimed for the repair of structural tissues should provide mechanical support in order to preserve tissue volume and ultimately to facilitate tissue regeneration. The most critical mechanical properties to be matched by the scaffold are bone loading stiffness, strength and fatigue strength. When the scaffold’s stiffness exceeds that of natural bone, stress concentration in the surrounding bone can cause bone failure. Conversely, when the scaffold’s stiffness is less than that of natural bone, stress concentration in the scaffold can cause implant failure as well as bone atrophy. This effect of stiffness mismatch, which gives rise to uneven load sharing between bone and implant, is known as stress shielding [46]. Stress shielding affects the bone remodeling and healing process. The underloaded bone adapts to the low stress environment and becomes less dense and consequently weak. In addition to matching bone stiffness, the scaffold should also match or exceed the strength of natural bone. The scaffold must resist physiological forces within the implantation site and should have sufficient strength and stiffness to function for a period until *in vivo* tissue ingrowth has filled the scaffold matrix. An equal or excess strength ensures that the scaffold has equivalent or better load bearing capabilities than natural bone. For last, for a non-resorbable scaffold, it is very important to consider the fatigue strength, since the scaffold will be exposed to cyclic loading during the rest of the patient’s life. Complete design of the scaffold must take into account both the mechanical considerations and the biological requirements to produce a globally optimized structure with an adequate chemical composition able to allow the subsequent ingrowth of bone.

In the scaffold design, surface properties including: topography, surface energy, chemical composition, surface wettability, surface bioactivity, etc., must all be considered, taking into account that in a complex porous 3-D scaffold the surface is not just the outside surface, but also the internal 3-D surfaces. For example, the modification of scaffolds materials with bioactive molecules is a technique to tailor the scaffold bioactivity. In addition, reduction of micromotion can be obtained by appropriately tailoring the material surface of the scaffold. The development of the required interface is not only highly influenced by surface chemistry, but also more specifically by nanometer and micrometer scale topographies. The surface roughness is found to influence the cell morphology and growth. It has been proved that alteration in surface topography by physical placement of grooves and depressions changes the cell orientation and attachment [47,48]. In general, smooth surfaces exhibit less cell adhesion than rough surfaces. On the other hand, surface porosity is another important factor in bone replacement [49,50]. It has been reported that materials coated with a porous surface exhibit less fibrous capsule formation than bulk or non-porous materials [51].

Surface modifications, such as, immobilization of biofunctional polymers and biopolymers, calcium phosphate ceramic coatings, hybridization with biocompatible and essential biomolecules are needed to achieve the required tissue induction properties. Countless procedures have been developed to modify the surface of biomaterials. Table 2 shows an overview of the surface modification methods available for titanium and its alloys. It has been widely demonstrated that surface treatment of titanium and its alloys has a critical influence on biocompatibility.

**Table 2:** Surface modification techniques available for pure titanium and titanium alloys. Modified from Liu X. *et al.* [52].

	Surface modification techniques	Modified layer	Purpose
**Mechanical Methods**	Machining / Electrochemical micromachining (EMM) Grinding Mechanical polishing Polishing media: SiC, Al_2_O_3_, diamond Grit-blasting e.g. Al_2_O_3_, SiO_2_, ZrO_2_, TiO_2,_ etc.	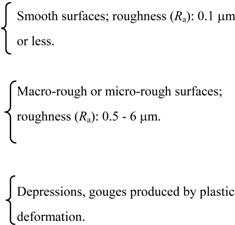	All the mechanical methods are able to produce a good surface finish, alter the native oxide layer and generate specific topographies leading to improve the biological fixation.
**Physical** **Methods**	Physical vapor deposition (PVD) Evaporation Ion plating Reactive sputtering	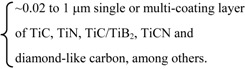	Decrease erosive and abrasive wear rates, improve corrosion resistance and improve hemocompatibility.
	Ion implantation and deposition Plasma immersion ion implantation and deposition (PIII&D) Beam-line ion implantation Glow-discharge plasma treatment (GDP)	< 50 nm of surface modified layer. < 150 nm of surface modified layer. < 1500 nm of surface modified layer. 20 nm to 2 μm of surface modified layer.	Modify the surface composition by incorporation of ionic groups improving the surface bioactivity and bone conduction. Surface topography can be altered. Improve wear and corrosion resistance. 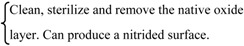
	Thermal spray Flame spray (FLSP) Plasma arc spray (PSP) High velocity oxygen fuel (HVOF) Detonation gun (D-Gun) Electric arc spray (EASP)	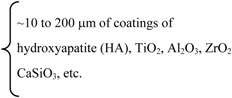	Modify the surface structure and composition. Improve wear and corrosion resistance and biocompatibility.
**Wet Chemical Methods**	Biomimetic method	Bone-like apatite precipitates are formed from a simulated body fluid (SBF), <50 μm	Improve biocompatibility.
	Acid etching e.g. HNO_3_-HF, HCl, H_2_SO_4_ Alkali-and heat-treatment Dual treatment (acid + alkaline) Hydrogen peroxide treatment	< 10 nm of surface modified layer. ~ 1 μm NaTiO_3_ hydrogel layer. ~ 10 nm surface layer containing hydrated oxide, peroxide and superoxide.	Remove native oxide film and contamination. Produce sub-micron porosity Induced roughness on a micrometer scale, enhance the biomimetic coating capacity and the bone-bonding ability Clean the surface, alter surface topography and induce bioactivity to the surface.
	Sol-gel process	~30 μm homogeneous and adherent thin films of TiO_2_, CaPO_4_, SiO_2_, etc.	Thicker and denser films with a special topography to improve the biocompatibility.
	Electrophoresis Electrophoretic deposition (EPD)	< 10 μm of surface modified layer.	Induce bioactivity to the surface, improve biocompatibility.
	Immobilization of functional groups (i.e. -SO_3_H, -PO_4_H_2_, -COOH via SAMs to Ti )	~10 μm to 15 μm of calcium phosphate films.	Induce bioactivity to the surface, improve biocompatibility.
	Electrochemical anodic oxidation Anodic spark deposition (ASD)	~10 nm to 50 μm of TiO_2_ layer, with the adsorption and/or incorporation of ions (S, P, or Ca/P). Nano to micrometer TiO_2_ layer.	Increases the thickness of the oxide layer producing a micro porous structure. Improves bioactivity, and corrosion resistance.
**Chemical Methods**	Thermal oxidation	~20 nm to 1 μm of TiO_2_ layer.	Produces a thick layer of TiO_2_ with a morphologically rugged surface.
	Chemical vapor deposition (CVD) Plasma-enhanced CVD	~1 to 5 μm single or multi-coating layer of TiO_2_,TiO_x_, TiC, TiN, TiCN and diamond-like carbon, among others.	Decrease erosive and abrasive wear rates, improve corrosion resistance and improve hemocompatibility.

SAMs: Self-assembled monolayers.

On the other hand, to program scaffolds with biological structures, cells and growth factors need to be integrated into the scaffold fabrication for bone tissue engineering, so that the bioactive molecules can be released from the scaffold in order to stimulate or modulate new tissue formation. Through surface modifications the metallic scaffold surface can be tailored to improve the adhesion of cells and adsorption of biomolecules in order to stimulate the bone formation and to facilitate faster healing [53]. Currently, a significant research effort is aimed at the biochemical modification of metallic surfaces. Table 3 presents some of the biochemical methods for surface modification of scaffolds introduced in recent years. The goal of the biochemical surface modification is to immobilize proteins, enzymes or peptides on biomaterials for the purpose of inducing specific cell and tissue responses, or in other words, to control the tissue-scaffold interface with molecules delivered directly to the interface. Nowadays, for the regeneration of structural tissues the bone tissue engineering is focusing in the development of a common framework for designing and building porous structures having both materials and biological components.

**Table 3:** Biochemical surface modification methods available for bone scaffolds.

	Surface modification techniques	Modified layer	Purpose
**Biochemical** **Methods**	Modification through biological polymers: collagen, fibrin, peptides, alginates, chitosan, hyaluronic acid, etc.	~10 nm to 5 μm of surface modified layer.	Induce specific cell and tissue response, enhancing the osseointegration.
	Modification through synthetic polymers: PLA, PGA, PCL, PC, etc.	~10 nm to 1 μm of surface modified layer.	Used as carriers of growth factors for local drug delivery.
	Biochemical factors or other inductive signaling molecules or drugs incorporation	~10 nm to 1 μm of surface modified layer.	Stimulate fracture healing and bone mineralization.
	Autologous or allogenic bone marrow cells Autologous or allogenic platelet concentrate Mesenchymal stem cells Chondrocytes, etc.	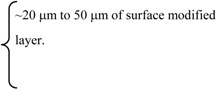	Impart osteogenic capacity by providing an environment that mimics that of the ECM.

PLA: polylactic acid; PGA: polyglycolic acid; PCL: polycaprolactone; PC: polycarbonate; ECM: Extracellular matrix.

In summary, the hybridization with active biofactors (cells, genes and/or proteins), the chemical composition, and the topography (structure, morphology) of the scaffold surface are known to be extremely important in bone replacement, since they regulate the type and degree of the interactions that take place at the interface: adsorption of ions and biomolecules such as proteins; formation of calcium phosphate layers; or interaction with different type of cells (macrophages, bone marrow cells, osteoblasts, etc.) [54,55]. For this reason, in recent years there has been a lot of research effort aimed at optimizing and controlling surface properties of the bone scaffolds with a view to customizing a certain material for the required application.

Equally important for the success of the loading-bearing scaffold is the postoperative stability. The scaffold and the surrounding bone must be tightly apposed to ensure osteointegration. In order to achieve stability over the time the scaffold must fulfill each patient anatomical requirement. Anatomically, the external geometry and size of the scaffold should be the same as those of the tissue defect in order for the scaffold to fit and anchor into the defect site. Computed-aided tissue engineering enables the application of advanced computer aided technologies and biomechanical engineering principles to derive systematic solutions for the designing and fabrication of patient-specific scaffold [56].

Finally, the scaffold for bone repair should be easy to manufacture with highly consistent pore sizes, pore distribution, pore density and interconnectivity with a narrow size distribution range of the structural parameters over the entire volume of the scaffold [57]. And for last, the scaffold must withstand sterilization procedures without loss of properties and have an acceptable shelf-life.

## 5. Currently Used Metallic Scaffolds Materials and Their Limitations

To date there are several biocompatible metallic materials that are frequently used as implanting materials in dental and orthopedic surgery to replace damaged bone or to provide support for healing bones or bone defects. Standard surgical implant materials include stainless steel 316 L (ASTM F138), Co based alloys (mainly ASTM F75, and ASTM F799) and titanium alloys; where Ti-6Al-4V (ASTM F67 and F136) are the most employed. However, the main disadvantage of metallic biomaterials is their lack of biological recognition on the material surface. To overcome this restraint, surface coating or surface modification presents a way to preserve the mechanical properties of established biocompatible metals improving the surface biocompatibility. Moreover, in order to enhance communication between cells, facilitating their organization within the porous scaffold; it is desired to integrate cell-recognizable ligands and signaling growth factors on the surface of the scaffolds. Indeed, biofactors that influence cell proliferation, differentiation, migration, morphologies and gene expression can be incorporated in the scaffold design and fabrication to enhance cell growth rate and direct cell functions [58]. Another limitation of the current metallic biomaterials is the possible release of toxic metallic ions and/or particles through corrosion or wear possible that lead to inflammatory cascades and allergic reactions, which reduce the biocompatibility and cause tissue loss [59]. A proper treatment of the material surface may help to avoid this problem and create a direct bonding with the tissue.

On the other hand, depending on the materials properties, some metallic materials are too weak to be arranged into the desired architecture with a controlled porous structure and some metals are too stiff and would fracture when arranged into certain architectures. Each metallic material possesses different processing requirements and the degree of processability of each metal to form a scaffold is variable also.

### 5.1. Tantalum

Porous tantalum is a biomaterial with a unique set of physical and mechanical properties. It has a high-volume porosity (>80%) with fully interconnected pores to allow secure and rapid bone ingrowth [60]. In addition, it has a modulus of elasticity similar to that of bone, which minimizes stress-shielding. Porous tantalum is a structural material and has sufficient strength to allow physiological load-carrying applications and represents an alternative metal for primary and revision total knee arthroplasty (TKA) with several unique properties. Bobyn and coworkers [60,61] presented basic scientific data that lend support for the use of this material, which is a trabecular metal composed of a carbon substrate that has elemental tantalum deposited on the surface. This trabecular metal has been shown to be highly biocompatible in several animal models [60,61,133]. Studies have demonstrated substantial cortical bone ingrowth between the trabecular network as well as high levels of bone growth onto the scaffold itself. Initial stability of the trabecular metal itself is also higher than that of standard materials, such as cobalt chrome. Furthermore, this new material offers better osteoconduction than other technologies used for biological fixation. Although porous tantalum is in its early stages of evolution, the initial clinical data [135,136,137,138] and preclinical studies [178,179,180,181,182,183,184,185] support its use as an alternative to traditional orthopedic implant materials.

### 5.2. Magnesium

The use of magnesium and its alloys for surgical applications is of particular interest. These alloys have great potential, and it has been shown that they are fully bioresorbable, have mechanical properties aligned to bone, induce no inflammatory or systemic response, are osteoconductive, encourage bone growth, and have a role in cell attachment [62]. Furthermore, because of its biodegradability, the second surgery for the removal of the scaffold might be avoided. All these facts suggest that Mg has significant potential as a load-bearing biomaterial. Indeed, there is a renewed interest in the use of this material in biomedical applications, e.g. for coronary stents [63,64], and more recently, researchers have concentrated on the application of magnesium-rare-earth alloys with new elemental contributions of cerium, neodymium and praseodymium for bone fixation devices [65,66] for osteo-applications. Recently, Mg-Ca alloys have been also produced and evaluated *in vitro* and *in vivo* as biodegradable biomaterials for orthopedic applications [67]. However, concerns over the toxicity of dissolved Mg have been raised, but it has been shown that the excess of magnesium is efficiently excreted from the body in urine [68]. In addition, concern does remain over the use of pure Mg as the dissolution rate in physiological conditions is rapid, potentially leading to hyper-magnesia, although a number of potential routes to controlling the corrosion rate have been proposed; especially providing it with a ceramic coating [69], titanium coating [70] or through the use of Mg alloys, including AZ31, AZ91, WE43, LAE442 and Mg-Mn-Zn alloys [65,66,71]. Although limited long-term survival data is available for Mg or Mg alloys porous scaffolds, the material seems promising for certain bone ingrowth applications such as trabecular bone regeneration.

### 5.3. Titanium and Titanium Alloys

Titanium is found to be well tolerated and nearly an inert material in the human body environment. In an optimal situation titanium is capable of osseointegration with bone [72]. In addition, titanium forms a very stable passive layer of TiO_2_ on its surface and provides superior biocompatibility. Even if the passive layer is damaged, the layer is immediately rebuilt. In the case of titanium, the nature of the oxide film that protects the metal substrate from corrosion is of particular importance and its physicochemical properties such as crystallinity, impurity segregation, etc., have been found to be quite relevant. Titanium alloys show superior biocompatibility when compared to the stainless steels and Cr-Co alloys. Titanium-aluminum-vanadium alloys (ASTM F136, ASTM F1108 and ASTM F1472) have better mechanical properties than commercially pure titanium (cp Ti) (ASTM F67) and are used more widely in total joint implants. However, concerns have been expressed about the presence of long-term Ti-6Al-4V implants, because elements such as vanadium are toxic in the elemental state. These concerns have led to the development of new beta titanium alloys with non-toxic alloying elements like Ta, Nb, Zr [73]. Other currently available titanium alloys include ASTM F1295 (wrought Ti-6Al-7Nb alloy), ASTM F1713 (wrought Ti-13Nb-13Zr alloy), ASTM F1813 (wrought Ti-12Mo-6Zr-2Fe alloy) and ASTM F2066 (wrought Ti-15Mo alloy) and Ti-5Al-2.5Fe (ISO 5832-10). Further biocompatibility enhancement and lower modulus has been achieved through the introduction of second generation titanium orthopedic alloys including Ti-15Mo-5Zr-3Al, Ti-15Zr-4Nb-2Ta-0.2Pd, Ti-12Mo-6Zr-2Fe, Ti-15Mo-3Nb-3O and Ti-29Nb-13Ta-4.6Zr. This new generation of Ti alloys is at present under development and investigation, and it does not seem to be commercialized yet. In general, porous titanium and titanium alloys exhibit good biocompatibility. Bioactive titanium meshes have been successfully used in spine fusion surgery for the past two decades [74]. The titanium mesh cage contoured into cylindrical shape has been used successfully for anterior lumbar interbody fusion (ALIF) for more than 15 years in surgery. Titanium mesh cages were also used with autografts for bone grafting in spinal fusion. This is restricted by factors such as complications and second site morbidity [74]. One method to overcome this problem is the use of hydroxyapatite to provide the necessary bioactivity to the titanium mesh cage with a porous network to facilitate osteoconduction [196,199]. Moreover, despite the great advances in complete tissue engineered oral and maxillofacial structures, the current gold standard for load bearing defect sites such as mandible, maxilla and craniofacial reconstruction remains titanium meshes and titanium 3-D scaffolds. On the other hand, Ti and its alloys are not ferromagnetic and do not cause harm to the patient in magnetic resonance imaging (MRI) units. Titanium osseointegration can be potentially improved by loading the scaffold with specific growth factors. In applications where there are existing gaps, such as craniofacial reconstruction or augmentation of bone or peri-implant defects, increased regeneration of bone, often has been accomplished with delivery of TGF-β and BMP-2 via titanium scaffold [30,75]. The latter growth factors are capable to elicit specific cellular responses leading to rapid new tissue formation. Stem cells have also been cultured *in vitro* onto titanium scaffolds [76] to induce the formation of calcified nodules in order to increase the production of mineralized extracellular matrix (ECM) onto the cells/scaffold constructs.

### 5.4. Nickel-Titanium Alloy (Nitinol)

Nitinol is one of the most promising titanium implants that find various applications as it possesses a mixture of novel properties, even in a porous state, such as shape memory effect (SME), enhanced biocompatibility, superplasticity, and high damping properties [77,78]. Since the elastic modulus of the Nitinol foams (*~*2.3 GPa) and the compressive strength (~ 208 MPa) are close to that of the bone and due its good biocompatibility porous NiTi have been used in making intramedullary nails and spinal intervertebral spacers used in the treatment of scoliosis [79]. Extensive *in vivo* testing and preclinical experience indicates that Nitinol is highly biocompatible, more than stainless steels [79,80]. Moreover, good biocompatibility on surface modified NiTi has been reported [81,82,83,84]. The demonstrated biocompatibility of Nitinol, its physical properties and SME, suggest that this alloy may offer substantial gains in the orthopedic field. These gains revolve around creating scaffolds that change shape after implantation due to the SME of Nitinol that can be initiated at the temperature of the human body. However, there is a problem of allergy and toxicity for NiTi alloys associated with the release of Ni ions. The concern of Ni toxicity and potential carcinogenicity has limited the use of NiTi alloys in Europe and the USA. In order to overcome this problem, surface modifications such as oxidation treatment of NiTi to obtain a Ni-free surface [85] and several alternative Ni-free shape memory alloys, mainly Nb-based, are currently under development although their long-term biological performance will have to be assessed in the future [86].

### 5.5. Hybrid Materials

Hybrid materials are those in which more than one class of material is employed in the scaffold. Today there are many different types of materials combinations principally used in artificial joints and bone implants. Many combinations of materials and surface modifications are aimed to stimulate specific responses at the molecular level. The synergistic combination of two types of materials may produce new structures that possess novel properties.

Common material combinations are synthetic polymer with bio-ceramic and synthetic/natural polymers with metals. Novel metal-ceramic-polymer hybrid materials have also been proposed for the fabrication of load-bearing scaffolds. In many clinical cases, composite scaffolds may prove necessary for reconstruction of structural diseases and bone defects. Nevertheless, the mechanical property requirements for hard tissue repair are difficult to satisfy using porous polymer/ceramic composites. Particularly, scaffolds based on HA or tricalcium phosphates (TCP) are very stiff, maybe brittle and may have different viscoelastic properties from bone [87]. To assure the mechanical integrity, hybrid constructs of porous Ti/TCP ceramic and cells have been tried and have demonstrated better osteogenic properties compared with Ti scaffold alone after implantation in goats [150]. Porous Ti is usually combined with bone inductive materials or cells, which endow the osteoinductive property leading to a rapid bone healing.

## 6. 3-D Metallic Scaffolds Fabrication Technologies

Numerous fabrication techniques have been developed for the production of 3-D metallic scaffolds of high porosity and surface area for load-bearing applications. The basic goal of the available manufacturing techniques is to produce a micro-architecture in a scaffold that is highly porous to allow for cell adhesion, vascularization and nutrient flow. Mechanical considerations however, limit the range of porosities at the optimum pore size that can be employed to produce functional structures. Strength and ductility of porous structures are very sensitive to final density, pore size, material type, and fabrication parameters. Metallic scaffolds can be produced in a variety of ways, using conventional techniques or advanced processing methods. The choice of the technique depends on the requirements of the final application. Selection of the scaffold material and design, the method by which to construct them, and the possible additional surface modification are important to the success of using the scaffold to regenerate new bone.

### 6.1. Conventional Fabrication Methods

Conventional methods for manufacturing metallic scaffolds include sintered metal powders [88], sintered metal fibres [89], space-holder method [90], replication of polymeric sponge [91], fiber meshes and fiber bonding [92], self propagating high temperature synthesis (SHS) [93], spark plasma sintering (SPS) [94] or field assisted consolidation technique (FAST) [95], gas injection into the metal melt [96], decomposition of foaming agents [97,98,99], templated vapor deposition [60] and solid-state foaming by expansion of argon-filled pores [100]. However, there are inherent limitations in these processing methods, which offer little capability to control precisely pore size, pore geometry, pore interconnectivity, spatial distribution of pores, porosity, etc. As a result, there are really few manufacturing technologies capable of producing porous structures that possess the majority of the desired requirements. Moreover, the manufacturing of porous titanium and its alloys is associated with some difficulties; most notably the extreme chemical affinity of liquid titanium to atmospheric gases such as oxygen, hydrogen, and nitrogen, which eventually leads to strongly reduced ductility [101]. Table 4 shows a comparison between the different conventional fabrication methods that have been applied to produce metallic porous structures.

**Table 4:** Comparison of various conventional fabrication methods for manufacturing metallic porous scaffolds. Numbers in parentheses correspond to the typical porosity (ε) values that each method is able to achieve. Modified from Ryan G. *et al.* [100].

	Closed-cell porosity		Open-cell porosity	
Random pore distribution		Porosity gradient^*^	Non-homogeneous	Homogeneous
Gas injection into the metal melt [96] ( ε =10-75%)		Spark plasma sintering (SPS) [94] ( ε = 50-60%)	Sintered metal powders [88] ( ε =20-90%)	Fiber meshes sintering [92] ( ε ≤ 90%) Fiber bonding [173] ( ε ≤ 70%)
Decomposition of foaming agents	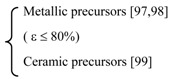 ( ε = 40-80%)	Field assisted consolidation technique (FAST) [95] ( ε = 50-60%)	Sintered metal fibers [89] ( ε =20-80%)	Templated vapor deposition [60] ( ε = 80-95%)
Gas entrapment [100] ( ε = 45-55%)			Space-holder method [90] ( ε ≤ 70%)	
			Replication [91] ( ε =80-95%)	
			Self propagating high temperature synthesis (SHS) [93] (ε ≤ 50%)	

^*^ Larger pores near the surface and smaller pores far form the surface.

The porous structure of the closed-cell structures is equiaxed and pores are surrounded by a metallic wall. In contrast, open-cell structures incorporate interconnected pores. Porous metals with elongated pores aligned in one direction—lotus structures—have recently been described also [96,98]. Scaffolds fabricated using conventional technologies have been employed clinically. Sintered bead coatings have been developed commercially using cobalt chrome and titanium alloys and have been shown to produce a durable biological bond that may last over ten years post implantation [103,104,105,106]. Diffusion bonded fiber-mesh porous structures have also been shown to successfully promote long-term implant fixation [107,108,109,110]. However, the maximum porosities attainable using these technologies is less than 50% at the required 100-700 μm pore sizes [111].

### 6.2. Rapid Prototyping (RP) Technology

In early 1980s, rapid prototyping technology emerged in the hi-tech manufacture industry [112]. Since this technique can fabricate products with complex structure and individuation at a small batch, it can be realized in one design-manufacture process with high flexibility. Products with different shapes can be obtained by only modifying the computer-aided design (CAD) model using 3-D tomography data or magnetic resonance imaging (MRI) data, shortening the production cycle. The digital information is then converted to a machine specific cross-sectional format, expressing the model as a series of layers. The file is then implemented on the RP machine, which builds customer designed 3D objects by layered manufacturing strategy. Each layer represents the shape of the cross-section of the model at a specific level. Conventional manufacturing methods [111] are either difficult to employ or are unsuccessful in producing such porous devices with complex structure with the tight constraints of porosity, optimum pore size, or mechanical strength that are required. The drawbacks of the traditional methodologies for producing porous constructs include long fabrication periods, labor-intensive processes, incomplete removal of residual chemicals or volatile porogenic elements, poor repeatability, irregularly shaped pores, insufficient interconnectivity of pores and thin wall structures, etc. RP techniques, also variously called solid free-form fabrication (SFF) or rapid manufacturing (RM), are considered a viable alternative for achieving extensive and detailed control over the scaffold architecture, shape and interconnectivity [113].

RP systems can also be utilized to produce a sacrificial mould to fabricate scaffolds. The multistep method involves casting of material in a mold and then removing or sacrificing the mold to obtain the final scaffold. Another important biological requirement is the surface properties of the fabricated scaffold. The topography of rapid prototyped surfaces can be further modified by sandblasting, shot-peening, vibratory deburring, spark anodization, electropolishing, acid etching, etc. Taking advantage of the possibilities of RP techniques load-bearing scaffolds with any predesigned structure and mechanical properties can be produced; so that they mimic the properties of the native bone and possess suitable strength for the intended application. Furthermore, CAD enables computational modeling and finite element analysis (FEA) prior to fabrication. Fluid flow analysis or stress distribution profiles can be obtained from computational models, thus allowing for re-design and scaffold optimization with minimal effort.

Until now RP developments mainly focused on polymer and ceramic materials [114]. However, recently several investigations have been carried out in order to produce 3-D porous metallic scaffolds using the RP route from 3-D solid models produced in CAD. For example, Li *et al.* [115] used a RP technology called 3D fiber deposition (3DF) for the fabrication of porous Ti-6Al-4V scaffolds with fully interconnected porous network and highly controllable porosity and pore size. Curodeau *et al.* [116] produced porous CoCr scaffolds manufactured by sacrificial wax template or investment casting. Murr *et al.* [117] reported the direct metal fabrication of non-stochastic titanium structures by electron-beam melting (EBM). Mullen *et al.* [118] produced porous titanium constructs by selective laser melting (SLM). This group also demonstrated that optimized structures can be produced with ideal qualities for bone ingrowth applications.

**Table 5:** Comparison of the different rapid prototyping (RP) technologies available for the fabrication of scaffolds aimed for hard tissue replacement.

RP Technology	Material	Advantages	Disadvantages	Refs.
3-dimensional printing^TM^ (3DP)	Stainless steels, CoCr alloys, Ti and its alloys.	Microporosity induced in the scaffold; enhanced range of materials can be used; fast processing; Independent control of porosity and pore size.	Material must be in powder form; powdery surface finish; may required post-processing.	[116]
Sacrificial wax template	Ta, Ti and its alloys.	Less raw material required; the original properties of the material are well conserved.	Multisteps involved.	[120,175,153]
3D fiber deposition technique (3DF)	Ti and its alloys.	Preparation time is reduced; high surface quality and high dimensional accuracy shrinkage.	Material must be in powder form;low resolution.	[115]
Electron beam melting (EBM)	Ti and its alloys.	Fast speed and less total time required.	Costly; low surface quality and low dimensional accuracy shrinkage.	[117,121]
Selective laser melting (SLM)	Stainless steels, CoCr alloys, Ti and its alloys, intermetallics, refractory metals, high temperature alloys.	Large variety of materials can be used in the form of powder; does not use binders or fluxing agents.	Difficulty of removal of the unbounded powder from the porous internal architecture; costly.	[118,119]
Direct metal deposition (DMD)	Ti and its alloys.	Deposit metals directly by layer deposition without patterns; good geometry control and surface finish.	Material must be in powder form; multisteps involved	[122]
Laser-engineered net shaping (LENS ^TM^ )	Stainless steels, CoCr alloys, Ti and its alloys, intermetallics, refractory metals, high temperature alloys.	Reduce the lead time and investment cost for modules and dies.	Material must be in powder form; costly.	[123,124]
Selective laser sintering (SLS)	Stainless steels, Ti and its alloys.	When mixed powders are used the powder of low melting point act as a binder; very fine resolution can be achieved; versatile in lay-down interconnected porous design.	Material must be in powder form; powdery surface finish; post-processing is required to increase the final density and mechanical properties.	[125, 166]

Table 5 summarizes the key features of several RP techniques commonly used for the fabrication of porous metallic scaffolds. In general, the dimensional accuracy, mechanical properties, and applicable materials are restricted by each particular technology. Future development of porous constructs is mainly concerned with improving the RP techniques for creating specialized, low costs structures, which give long-term mechanical reliability to the engineered porous metal-bone interface.

## 7. Biocompatibility of Commercially Available Metallic Scaffolds

### 7.1. Tantalum

The efficacy of tissue-engineered tantalum constructs has been tested extensively in preclinical and clinical trials. Table 6 and Table 7 show respectively the results of some preclinical and clinical trials using porous tantalum scaffolds.

**Table 6:** Preclinical studies using Ta scaffolds.

Author	Animal model & implantation site	Clinical results	Demonstrated properties
Zhang, Y. *et al.* [132] (1999)	Implantation of Ta porous scaffold in bovine cortical bone in order to investigate the interfacial frictional characteristics.	The coefficient of friction of porous Ta was higher than the coefficient of friction of cortical or trabecular bone.	Ta porous scaffold exhibits a high friction coefficient.
Bobyn, J.D. *et al.* [60] (1999)	Implantation of Ta porous scaffold using a transcortical canine model.	By 16 and 52 weeks the average extent of bone ingrowth ranged from 63% to 80%. A max shear strength fixation of 18.5 MPa was obtained.	The tantalum construct allowed extensive bone ingrowth exhibiting high fixation strength at all the implantation periods.
Bobyn, J.D. *et al.* [61] (1999)	Implantation of porous Ta components in the femora of dogs.	Thin section histology revealed that the implants had stable bone-implant interfaces after 6 months.	The Ta components exhibited adequate porous architecture to allow bone ingrowth.
Hacking, S.A. *et al.* [133] (2000)	Subcutaneous implantation of porous Ta scaffold into the back muscle of dog.	Fibrous tissue ingrowth and blood vessels progressively increased during the first 8 weeks after which this increase leveled off.	Inside the Ta scaffold architecture normal fibrous ingrowth and high attachment strength was observed.
Rahbek, O. *et al.* [134] (2005)	Implantation of porous Ta components in the into the knee joints of dogs for 8 weeks. Weekly polyethylene (PE) particle injection into the knees was done to determine the resistance to migration of PE particles.	Porous Ta exhibited superior bone ingrowth, more bone marrow, less fibrous tissue and less PE particle migration compared with glass bead blasted Ti.	Porous Ta showed resistance to migration of PE particles and superior bone formation.
Adams, J.E. *et al.* [135] (2005)	Implantation of a cylindrical dowel of porous Ta (Zimmer, Warsaw, Indiana) into a defect created at the junction of the radial carpal bone, the ulnar carpal bone, and the forth-carpal bone of canines.	Histology showed bony ingrowth as early as 4 weeks and mechanical testing showed a statistically significant increase in strength of the construct over time.	The porous Ta served as an adjunct to stabilization of the carpus in the canine model of four-corner fusion.
Zou, X. *et al.* [136] (2004)	Implantation of a porous Ta solid piece, porous Ta ring (Hedrocel®) packed with autograft and as a control a carbon fiber cage in a porcine lumbar interbody fusion (ALIF) model.	Bone ingrowth was observed after 3 months of implantation with no significant difference between the porous Ta and porous Ta ring and the carbon fiber cage.	The radiographic and histological appearance of the porous Ta ring was equivalent to the carbon fiber in the porcine ALIF model.
Tanzer, M. *et al.* [137] (2001)	Bilateral implantation of porous Ta intramedullary cylindrical rods (mean pore size 430 mm, volume porosity between 75-80%) into the ulnae of dogs. One leg was treated with ultrasound and the other acted as a control.	Bone ingrowth was observed in both legs; however, 119% more bone ingrowth was obtained into the ultrasound treated leg compared with the contralateral control.	Non-invasive low intensity ultrasound may provide a reliable, safe and inexpensive modality to augment bone ingrowth into cementless arthroplasties of all designs.

**Table 7:** Clinical studies using tantalum scaffolds.

Author	Animal model & implantation site	Clinical outcomes	Demonstrated properties
Meneghini, R.M. *et al.* [138] (2008)	Implantation of porous tantalum metaphyseal cones（Zimmer Inc. Implex, USA）into 15 patients with total knee replacement (average age of 68.1 years).	The average Knee Society clinical scores improved form 52 points preoperatively to 85 points after 34 months. All the cones showed evidence of osseointegration with reactive osseous trabeculation at points of contact with the tibia.	Porous tantalum metaphyseal cones effectively provided structural support for the tibial implants in this study.
Long, W. *et al.* [139] (2009)	Implantation of porous tantalum metaphyseal cones（Zimmer Inc. Implex, USA）into 16 patients with total knee arthoplasty.	2 cases of recurrent infection occurred. The remaining 14 cases were functioning well during the average 31 months follow up.	Porous cones were found to be well fixed with stable bony ingrowth. The porous cones are a better alternative than placing large amounts of dead bone or large metal augments into the defect.
Nadeau, M. *et al.* [140] (2007)	Implantation of a porous Ta plug (Zimmer, Warsaw, Indiana) into 15 patients (average age of 42 years) with osteonecrotic hips with Steinberg stage III and IV.	The success rate at 12 months postoperatively was 77.8%, and the overall success rate was 44.5%. On average, patients who did well improved their Harris hip scores by 21.7 points.	Core decompression with porous Ta showed encouraging success rates in patients with advanced stage osteonecrosis, but further larger scale studies are required.
Tsao, A.K. *et al.* [141] (2005)	Implantation of a porous Ta plug (Zimmer, Warsaw, Indiana) into 98 patients (average age of 43 years) with early-stage hip osteonecrosis.	The average Harris hip score for all stage-II hips was 63 preoperatively, and after 4 years increased to 83. The survival rate was 72.5% at 48 months.	Initial stability was achieved with the threaded end of the scaffold and its reduced elastic modulus reduced abnormal stresses in the surrounding bone.
Durham, S.R. *et al.* [142] (2003)	Implantation of tantalum mesh for the repair of large (>25 cm^2^) cranial defects into 8 patients (1.5 to 35 years). The reasons for cranioplasty included cranial defect from trauma, fibrous dysplasia, infected bone flaps and tumor.	2/8 cranioplasty got infected and had to be removed at 1 and 3 months postoperatively.	The tantalum mesh used with HA cement and fixed with Ti plates provided internal structural support and increased the stability of the construct.
Shuler, M.S. *et al.* [143] (2007)	Implantation of a porous Ta plug (Zimmer, Warsaw, Indiana) into 24 patients (average age of 43.2 years) with early-stage hip osteonecrosis.	The survival rate was 86% (3 implants failed) at an average follow-up of 39 months. All the survivors were rated with the Harris hip score as good (14%) and excellent (72%).	The porous Ta scaffold is a safe option for femoral head salvage. Continued follow-up is necessary to determine the long term success of the clinical procedure.

HA: Hydroxyapatite.

Valuable preclinical results in laboratory animal experiments using commercially available tantalum constructs have led to the development of further applications of porous tantalum. For example, in total hip arthroplasty, spinal fusions, structural support of osteonecrosis and tumor related lesions, hand surgery lesions, maxillofacial surgery, etc. Data gained from these experiments have been invaluable leading to the advances of clinical trials in a controlled fashion. The majority of these short-term clinical studies exhibited promising favorable results, but long-term studies are needed. Nowadays, porous tantalum (Trabecular Metal™) *in vivo* testing is undergoing phase III and phase IV clinical trials.

### 7.2. Magnesium

Magnesium and magnesium alloys have similar mechanical properties with natural bone, but their high susceptibility to corrosion has limited their application in orthopedics. In the case of biodegradable scaffolds, it is desirable for the scaffold materials to be biodegraded completely after an appropriate period in a human body. An important method to slow down the degradation rate of magnesium is surface modification. Some surface modifications have been developed for porous Mg constructs to control the degradation rate as well as to improve the biocompatibility [126,127].

**Table 8:** Preclinical studies using magnesium scaffolds.

Author	Animal model & implantation site	Clinical results	Demonstrated properties
Reifenrath, J. *et al.* [144] (2005)	Implantation of magnesium alloy AZ91 open porous scaffolds (pore size distribution 10-1000 μm and 72-76% porosity) into the medial condyle of the knee of rabbits.	Osteoconductive properties in the rim of the scaffold were observed, however the material did not induce the formation of subchondral bone necessary for osteochondral defect repair.	AZ91 scaffold is a fast degrading material that cannot sufficiently replace the subchondral bone plate during the first 12 weeks of cartilage repair.
Witte, F. *et al.* [145] (2006)	Implantation of magnesium alloy AZ91 open porous scaffolds (pore size distribution 10-1000 μm and 72-76% porosity) into the patellar cartilage of rabbits.	New bone formation was observed at the rim of the degrading scaffold.	The surrounding cartilage tissue was not negatively affected by the rapid degradation process of the scaffold.
Witte, F. *et al.* [146] (2007)	Implantation of magnesium alloy AZ91D open porous scaffolds (pore size distribution 10-1000 μm and 72-76% porosity) into the distal femur condyle of rabbits to evaluate the inflammatory response.	After 3 months the scaffolds largely degraded and most of the magnesium alloy disappeared causing no harm to the neighboring tissues	Good biocompatibility with an appropriate inflammatory host response was observed.
Witte, F. *et al.* [147] (2007)	Implantation of magnesium alloy AZ91D open porous scaffolds (pore size distribution 10-1000 μm and 72-76% porosity) into the condyles of the knee of rabbits to evaluate the peri-implant bone remodeling.	Higher BV/TV and more mature bone structure were observed on the tissue surrounding the magnesium scaffolds compared with the control, which was autologous bone.	Fast degrading Mg scaffold induced extended peri-implant bone remodeling with a good biocompatibility.

BV/TV: Bone volume per tissue volume.

Nowadays only *in vitro* [126,127] and preclinical studies using animal models have proposed the usage of Mg scaffolds as degradable scaffolds for bone substitute applications. Indeed, works dealing with the *in vivo* behavior of porous magnesium at the preclinical level are still very scarce. Table 8 lists the data derived from some preclinical studies using magnesium or magnesium alloys constructs.

### 7.3. Titanium

Porous titanium and titanium alloys have been shown to possess excellent mechanical properties as permanent orthopedic implants under load-bearing conditions [128]. Many basic scientific preclinical and clinical studies support the utility of Ti scaffolds. For marginal bone defects and bone augmentation Ti foams allow for bone ingrowth through interconnected porous [155]. On the other hand, titanium fiber-mesh is a useful scaffold material that warrants further investigation as a clinical tool for bone reconstructive surgery. *In vitro*, titanium fiber-mesh acts as a scaffold for the adhesion and the osteoblastic differentiation of progenitor cells [129]. *In vivo*, the material reveals itself to be osteoconductive, demonstrating encouraging results [182]. The studies described in Table 9, performed in clinically relevant large animal models, provide a wealth data demonstrating the safety and feasibility of the use of titanium scaffolds in the healing of bone defects.

**Table 9:** Preclinical studies using Ti scaffolds.

Author	Animal model & implantation site	Clinical results	Demonstrated properties
Matsuzaka, K. *et al.* [148] (2005)	Implantation of Ti porous scaffold fabricated by space holder technique (pore size 200-500 μm, 78% porosity) with and without BMP-2 immobilization in rat femur.	Two weeks after implantation new bone tissue formed around the scaffold with and without BMP-2 immobilization.	Ti porous scaffold with BMP-2 can produce new bone tissue at an early stage and can be beneficial in the repair of bone defects.
Ponader, S. *et al.* [149] (2009)	Implantation of porous Ti6Al4V scaffold fabricated by selective electron beam melting (SEBM) (pore size 450 μm, 61.3% porosity) into defects in the frontal skull of domestic pigs.	Bone ingrowth (≈46%) was reached after 60 days and the healing bone structure in the outer region of the scaffold was comparable with that of pristine bone.	The scaffold shows adequate architecture to allow bone ingrowth and excellent mechanical properties.
Li, J.P. *et al.* [150] (2007)	Implantation of porous Ti6Al4V scaffold made by 3D fiber (3DF) deposition (pore size 160-680 μm, 39-68% porosity) into the posterior lumbar spine of goats.	Bone ingrowth progressively increased during the first nine weeks after which this increase leveled off.	Scaffold architecture can be easily controlled and changes in the porosity and pore size had a positive effect on the amount of new bone formation.
Bottino, M.C. *et al.* [151] (2009)	Implantation of powder metallurgy (P/M) processed Ti13Nb13Zr porous samples (pore size 50-100 μm, 30% porosity) into rabbit tibiae for 8 weeks.	Close bone-implant contact observed, however due to the absence of open as well as interconnected pores no bone ingrowth was observed.	Porous Ti13Nb13Zr manufactured by P/M with metallic hydrides were non-cytotoxic but pore structure and pore distribution were non appropriate for bone ingrowth.
Chang, Y.-S. *et al.* [152] (1998)	Implantation of fiber meshes fabricated by sintering and plasma spraying (pore size 200-400 μm, 56-60% porosity) into femoral defects in dogs.	Abundant bone ingrowth was observed that resulted in the complete integration of this composite device implant and the host bone.	Scaffolds with 3-D open pore structure led to complete osseointegration.
Lopez-Heredia, M.A. *et al.* [153] (2008)	Implantation of scaffold made by rapid prototyping (RP) technique (pore size 800 and 1200 μm, 60% porosity) into the femoral epiphysis of rabbits.	Bone ingrowth observed (≈24%) after 3 weeks with no difference between the two pore sizes. BIC were around 30%.	RP Ti scaffolds possess excellent mechanical and biological properties.
Takemoto, M. *et al.* [154] (2007)	Implantation of porous Ti with a bioactive titania layer fabricated by the spacer method (mean pore size 303 μm, 50% porosity) into the anterior lumbar spine in dogs.	Interbody fusion was confirmed in all five dogs. Histological evaluation demonstrated a large amount of new bone formation with marrow like tissue into the bioactive scaffolds.	Bioactive alkali and heat-treatment effectively enhanced the bone-bonding and the fusion ability of the porous Ti scaffolds.
Pinto-Faria, P.E. *et al.* [155] (2008)	Implantation of porous Ti sponge rods made by space holder method (pore size 200~500 μm, 80% porosity) for the healing of humerus bone defects in a canine model. As a control HA granules were used.	HA granules rendered more bone formation than the Ti foam after 2 and 4 moths of implantation. However the Ti foam led to a better bone-growth distribution in the implanted sites.	The Ti foam exhibited good biocompatibility, and its application resulted in improved maintenance of the bone height compared with control sites filled with HA granules.
Walboomers, X.F. *et al.* [156] (2005)	Implantation of hollow cylindrical fiber mesh scaffold filled and unfilled with COLLOSS® into the back of rats.	After 12 weeks of implantation in the control scaffold no bone-like tissue formation was evident in almost all samples.	The COLLOSS® filled scaffold showed bone-inducing properties. Bone marrow tissue formation was evident in almost all samples.

RBM: Rat bone marrow; BIC: Bone-implant contact; HA: Hydroxyapatite; COLLOSS®: Bovine extracellular matrix product containing native BMPs.

**Table 10:** Clinical studies using titanium scaffolds.

Author	Animal model & implantation site	Clinical outcomes	Demonstrated properties
van Jonbergen, H-P.W. *et al.* [157] (2005)	Implantation of titanium SynCage C (Synthes, Oberdorf, Switzerland) filled with autogenous bone graft into 71 patients (23 to 76 years) with cervical disc disease and cervical spinal stenosis.	Fusion was achieved after 6 months in all patients; however, 10 cages (each in a different patient) had subsided.	Subsidence behavior of this titanium cage deign was noted and is a disturbing phenomenon. A modified cage design with improved and extended lower contact surface could be expected to reduce subsidence.
Eck, K.R. *et al.* [158] (2000)	Implantation of titanium mesh cages into 66 consecutive adult patients (ages 20-81 years) with sagittal deformities. The cages were inserted into the anterior column during posterior instrumentation and fusion.	No cage failure or extrusion was observed. The average segmental improvement in lordosis with cage implantation was 11° with a loss of correction of less than 1° after 2 years.	Structural titanium mesh cages implanted into the anterior column functioned appropriately to maintain sagittal correction and with rare radiographic complications were obtained.
Kuttenberger, J.J. *et al.* [159] (2001)	Implantation of laser-perforated titanium micro-mesh (Howmedica Leibinger GmbH & Co., Germany) into 20 patients (ages 22-78 years) with defects in the craniofacial and/or orbito-ethmoidal region.	No wound infections, exposures or loss of the mesh have been observed. Long-term stability reconstruction was excellent (8 years follow-up).	Radiographs and CT scans demonstrated that stable 3-D reconstructions of complex anatomical structures were achieved in all the treated patients.
Bystedt, H. *et al.* [160] (2008)	Implantation of porous titanium granules (Natix ^TM^, Tigran Tech. AB, Sweden) into 16 consecutive patients (55 to 83 years) with the need of augmentation of the sinus floor.	1 patient had postoperative sinus infection. The postoperative radiographs showed no signs of migration of the granules.	Titanium granules seem to function well as augmentation material in the sinus floor. Biopsies to confirm bone ingrowth are needed.
Jaquiéry, C. *et al.* [161] (2007)	Implantation of titanium meshes some of them filled with autogenous bone graft into 26 patients (13 to 82 years) with small and mid-size orbital defects (categories I, II, and III).	Postoperatively, 91% of the patients had normal vision and accuracy of reconstruction was achieved in category II defects.	Titanium meshes provided stability and can support the orbital content preventing the risk of a secondary enophthalmos.
Scholz, M. *et al.* [162] (2007)	Implantation of individually prefabricated CAD/CAM titanium porous plate into 1 male patient (16-year-old) with a severe head injury including an intracranial hematoma.	CAD/CAM titanium porous plate served as a virtual template for a precise surgical resection along a pre-established geometry ensuring the perfect fit of the scaffold.	CAD/CAM titanium porous plate are suitable for reconstructing large bone defects in the skull because provide long-term stability, quick installation and very good cosmetic results. As a disadvantage, CAD/CAM technology is more expensive than a titanium mesh, and the process is time-consuming as it is carried out in advance of surgery.

CAD/CAM: Computer-aided design/computer-aided manufacturing.

Results of these preclinical studies confirm that healing of bone is possible using biochemically-modified Ti scaffolds, specifically by the use of growth factors and osteoprogenitor cells. However, the assessment of the potential for the use of these biochemically-modified Ti scaffolds for clinical applications in the future lies on the capability of the researches to show excellent long-term results. Clinically, cylindrical titanium meshes have been used with consistently good results for large anterior column defect reconstructions. Implantation of synthetic cages into the anterior column seems to offer immediately effective segmental stability, correction of the sagittal plane deformity, and restoration of the anterior vertebral support from a biomechanical standpoint. These anterior interbody cages provide a satisfactory axial load-bearing capacity, and morcellized autograft can be used to fill the inside of the cage [158]. Table 10 lists the results of some clinical studies that employed porous Ti scaffolds for hard tissue repairing and reconstruction. Although there is a paucity of literature regarding the clinical outcomes and result of porous titanium scaffolds, longer follow-up periods and a larger sample group of patients are required in order to obtain reliable clinical success rates.

### 7.4. Nickel-Titanium Alloy (Nitinol)

Porous Nitinol (PNT) has been used in maxillofacial and some orthopaedic surgeries in Russia and China for approximately 15 years [130]. PNT has aroused interest also in intervertebral disc pathologies as an interbody fusion bone scaffold [131]. However, until now few preclinical trials using animal models and very scarce clinical trials have been carried out and more research may be required to better understand the biological performance of PNT. Table 11 shows some preclinical studies carried out with porous Nitinol as scaffold material. The majority of the clinical studies using Nitinol meshes are limited to the thoracic and cardiovascular surgery field where Nitinol finds application especially in self-expanded metallic stents.

**Table 11:** Preclinical studies using NiTi scaffolds.

Author	Animal model & implantation site	Clinical results	Demonstrated properties
Ayers, R.A. *et al.* [163] (1999)	Implantation of NiTi porous scaffold fabricated by SHS (pore sizes 353, 218 and 179 μm, porosity 43, 54 and 51%) into cranial defects in rabbits.	Bone ingrowth observed in the three types of implants.	The used pore sizes appear not to affect bone ingrowth during the cartilaginous period of bone ingrowth.
Kujala, S. *et al.* [164] (2003)	Implantation of NiTi porous scaffold fabricated by SHS (pore sizes 259 and 505 μm, porosity 66 and 47%) into femoral defects in rats.	Bone ingrowth observed, porosity of 66% showed the best bone-implant contact.	The scaffold allows bone ingrowth, although fibrosis inside the porous structure was observed in some cases.
Simske, S.J. *et al.* [165] (1995)	Implantation of porous NiTi scaffold made by SHS (pore size ≈ 300 μm, ≈ 50% porosity) into cranial defects in rabbits.	Bone contact with the surrounding cranial tissue and bone ingrowth observed.	Porous NiTi exhibited more total bone ingrowth than coralline HA after 12 weeks of implantation.
Shishkovsky, I.V. *et al.* [166] (2008)	Implantation of porous NiTi scaffold made by SLS and SHS (nanostructured walls in the range of 1460-460 nm) into dextral blade bone of rats.	No adverse tissue reactions were observed and the histological samples showed no evidence of bone resorption in the cranial bone adjacent to the scaffolds.	The porosity and the surface chemistry engineered in the combined SLS-SHS process were suitable for biointegration.
Zhu, S.L. *et al.* [167] (2008)	Implantation of porous NiTi scaffold prepared by element powder sintering (mean pore size 130 μm, 45% porosity) into the long axis of the femur of rabbits.	Histological sections showed that the osteoblasts were directly in contact with the porous NiTi without intervenient fibrous tissue. Bone ingrowth was also observed in the inner of the scaffold.	Good bone-implant contact was obtained in the porous NiTi. Porous NiTi alloy exhibited better osteoconductivity and osseointegration than bulk one.
Rhalmi, S. *et al.* [168] (1999)	Implantation of porous NiTi blocks (5 × 3 × 3 mm volume, pore size range 400 μm < Ø < 900 μm) into the tibias and back muscle of rabbits.	Muscle tissue exhibited thin tightly adherent fibrous capsules with fibers penetrating into implant pores. Bone tissue demonstrated good healing of the osteotomy. There was bone remodeling characterized by osteoclastic and osteoblastic activity in the cortex.	Good biocompatibility acceptance of porous NiTi was observed in both muscle and bone tissue. The results corresponded very well with the *in vitro* cell culture evaluation.
Rhalmi, S. *et al.* [169] (2007)	Implantation of porous NiTi IFD manufactured by Biorthex Inc., Canada. (pore size 230 ± 130 μm, 65± 10% porosity) into the spinal canal of the dura mater at the lumbar level L2-L3 in rabbits.	In contact with the dura mater NiTi elicits an inflammatory response similar to that of Ti. The inflammation was limited to the spidural space and then reduced from acute to mild chronic after 1 year.	The tolerance of NiTi by a sensitive tissue such as the dura mater during the span of 1 year of implantation demonstrated the safety of NiTi and its potential use as an IFD.
Wu, S. *et al.* [170] (2008)	Implantation of a hydrothermally treated 3D porous NiTi scaffolds fabricated by CF-HIP into the femurs of rabbits. Hierarchical porous nanostructures external layer of bioactive titanate was obtained.	Bone tissue could grow smoothly into the internal pores of the scaffolds and made good contact with the exposed surface of the scaffold.	The external nanostructure obtained facilitates the biomineralization and promote deposition of bone-like apatite and proliferation of osteoblasts.

SHS: Self-propagating high-temperature synthesis; SLS: Selective laser sintering; IFD: Interbody fusion device; CF-HIP: Capsule free- hot isostatic pressing method.

Table 12 shows the results of two specific clinical trials using porous Nitinol. The first trial employed a porous Nitinol superelastic expandable cage that can be twisted into any shape and return to the original shape when the compression force is lifted [171]. The second trial corresponds to the midface endoprosthetic area. PNT constructs were used in the midface reconstruction in 129 patients with encouraging results [172]. However, continued follow-up is necessary to determine the clinical long-term success of these PNT constructs.

**Table 12:** Clinical studies using NiTi scaffolds.

Author	Animal model & implantation site	Clinical outcomes	Demonstrated properties
Wang, Y. *et al.* [171] (2008)	Implantation of NiTi porous superelastic cage in 62 patients (21 to 61 years) with total hip arthroplasty (THA).	The total survival rate was 82.7% (67/81 hips) without further treatment. Of 81 hips, 14 (17.3%) had progressive pain with collapsed femoral head resulting in THA.	The superelastic cage provided structural support to the subchondral bone in the necrotic femoral head, also decreased the further collapsing trend of the ONFH and helped to regain contour of articular surface of the collapsed femoral head.
Arsenova, I.A. *et al.* [172] (2005)	Implantation of porous NiTi scaffold saturated with bone marrow into midface bony defects into 129 patients (74 endoprosthetics of inferior wall of the orbit, 26 endoprosthetics of maxillary walls, 14 maxillary endoprosthetics, 12 endoprosthetics of supporting structures of the nose, 3 zygomoorbital region).	The study of grinds done after 180 days of implantation revealed that most part of the pores were filled with bone tissue, the quantity of calcium in the pores was similar to one in bone tissue. Positive results of endoprosthetics were achieved in 123 patients.	PNT structures possess good integration with tissue structures and need further study of possibilities of their use in reconstructive surgery of facial skull and temporomandibular joint (TMJ).

ONFH: Osteonecrosis of the femoral head; PNT: Porous Nitinol.

### 7.5. Hybrid Constructs

Upon the placement of a scaffold into a surgical site, there is a cascade of molecular and cellular processes that provides for new bone growth and differentiation along the biomaterial surface. The goal of a number of current strategies is to provide an enhanced osseous stability through micro-surface mediated events. These strategies can be divided into those that attempt to enhance the immigration of new bone (e.g., osteoconduction) through changes in surface topography (e.g., surface roughness, porous surface, etc.), biological means to manipulate the type of cells that grow onto the surface and strategies to utilize the scaffold as a vehicle for local delivery of a bioactive coating (adhesion matrix or growth factor such as BMPs) that may achieve osteoinduction of new bone differentiation within the scaffold surface. Calcium phosphate (Ca-P) ceramics have been successfully proposed as bone substitutes because of their chemical similarities with bone mineral. Hybrid constructs of Ti or Ta/osteogenic cells, Ti or Ta/Ca-P ceramics, and Ti or Ta/growth factors have demonstrated very good osteogenic properties compared with non-modified Ti or non-modified Ta, suggesting that the surface modification of Ti and Ta plays an important role in bone tissue engineering. In the case of Ca-P coated scaffolds physicochemical and crystallographic continuity have been observed *in vivo* between the calcium phosphate coated external surface and the newly mineralized layer [179]. This mineralized interface ensures a physicochemical and mechanical cohesion between the scaffold and the host bone. Table 13 and Table 14 show some preclinical trials made in different animal models using hybrid Ti and Ta constructs, respectively. In the field of tissue engineering, the grafting of arginine-glycine-aspartic acid (RGD) peptides has been the focus of much attention [176,181,185]. Their presence at biomaterials surfaces improved cell adhesion. Indeed, this peptide sequence is present in various extracellular matrix and plasma proteins, and it constitutes a major recognition site of a large number of adhesive extracellular matrix, blood and cell surface proteins [185]. The immobilization of bioactive molecules such as BMP-2 into the metallic biomaterial surface leads to the differentiation of the cells towards osteogenic lineage improving the osseointegration [174]. Based upon the promising results obtained from the preclinical studies, carefully selected and controlled clinical trials with rhBMP-2 have begun [201]. Ti scaffolds have also found application as delivery systems for transforming growth factors β-1 (TGF- β1) [183]. On the other hand, adjusting the underlying micro-and nanotopography is also a smart way to trigger and modulate specific cellular functions. This approach was attempted by Wu *et al.* [170] using porous NiTi, and Takemoto *et al.* [154] using porous Ti. The combined effect of topography and biochemical cues using bone-stimulating agents is indeed an interesting path to improve the biocompatibility.

**Table 13:** Preclinical studies using titanium-ceramic, titanium-polymer, or cell loaded titanium scaffolds.

Author	Animal model & implantation site	Clinical results	Demonstrated properties
Zhang, E. *et al.* [173] (2009)	Implantation of Si-HA coated-porous Ti fabricated by fiber sintering (pore sizes 150-600 μm, porosity 67%) into the femora of rabbits.	High bone ingrowth rate was observed inside the 3D interconnected pore structure.	Si-HA coating significantly improved the surface bioactivity of the porous Ti. The existent Si ions might have been the cause of the improved bioactivity obtained.
Peng, L. *et al.* [174] (2008)	Implantation of HA coated-porous scaffold fabricated by sintering premodified by alkali and heat treatment with BMP-2 and hylauronic acid into the femora of rabbits.	Bone ingrowth observed. HA-coated Ti scaffolds achieved lower osseointegration than the BMP-2 group.	HA-coated Ti scaffolds with BMP-2 and hyluronic acid had a good effect in repairing bone defects.
Lopez-Heredia, M.A. *et al.* [175] (2008)	Implantation of CaP-coated Ti scaffold made by rapid prototyping technique (pore size 1000 μm, 50% porosity) into the dorsal subcutaneous pounches of rats.	After 4 weeks of subcutaneous implantation mineralized collagen was observed but not mature bone.	Scaffold architecture could be easily coated with CaP and according to the *in vitro* evaluation with RBMC cells the biocompatibility was improved by the coating applied.
Sargeant, T.D. *et al.* [176] (2008)	Implantation of a Ti6Al4V foam made by HIPing (pore size 165 μm, porosity 52%), whose porosity was filled with a peptide amphiphile (PA) nanofiber matrix into a rat femoral defect.	PA-Ti hybrid constructs exhibited bone ingrowth and the newly formed bone around and inside the implant were highly mineralized after 4 weeks of implantation.	By filling the porosity of the scaffold with PA bone mineralization was successfully induced.
Sikavitsas, V.I. *et al.* [177] (2003)	Implantation of bone marrow stromal osteoblasts-loaded Ti fiber mesh composite scaffold (fiber Ø 45 μm, porosity > 86%) into calvarial defects in rats.	Bone ingrowth observed. The highest % of bone formation was obtained in the cell-loaded scaffolds (64%).	Osteoinductivity and high bone regeneration was achieved thanks to the cells loading.
Vehof, J.W. *et al.* [178] (2000)	Implantation of CaP-coated titanium fiber mesh (pore size 250 μm, 86% porosity) loaded with osteogenic cells into the back of rats.	None of the CaP-coated and non-coated meshes alone supported bone formation after 6 weeks. After 8 weeks bone formation was observed in CaP coated meshes.	The combination of Ti mesh with osteogenic cells can generate bone formation, and CaP has a beneficial effect on bone formation.
Habibovic, P. *et al.* [179] (2005)	Implantation of porous Ti6Al4V and OCP coated-Ti6Al4V produced by a positive replica technique (pore size 400-1300 μm, 79±5% porosity) into the femora and back muscle of goats.	OCP coated-Ti6Al4V showed a higher bone ingrowth and ectopic bone formation amount than uncoated Ti6Al4V.	OCP posses high osteoconductive potential. The coating was fully replaced by newly formed bone after 12 weeks.
Hartman, E.H.M. *et al.* [180] (2005)	Implantation of RBM cells loaded titanium fiber mesh (fiber Ø 50 μm, 86% porosity) and porous CaP into the back muscle in rats.	After 6 weeks limited bone ingrowth inside the cell-loaded Ti fiber mesh was found. The CaP group exhibited more bone formation.	RBM cell-loaded CaP is much more osteoconductive than RBM cell-loaded Ti fiber mesh.
Kroese-Deutman, H.C. *et al.* [181] (2005)	Implantation of RGD-loaded Ti fiber meshes (fiber Ø 45 μm, porosity > 86%) into the cranium of rabbits.	RGD-Ti scaffolds exhibited higher bone formation and bone ingrowth.	RGD in combination with Ti fiber mesh produces a positive effect on bone formation.
van der Dolder, J. *et al.* [182] (2003)	Implantation of RBM stromal cells loaded titanium fiber mesh (pore size 250 μm, 86% porosity) into cranial defects in rats.	RBM cells enhanced the initial bone formation and union of the skull bone with bone inside the Ti fiber mesh only occurred in the cell-loaded scaffolds.	Bone compatibility of cell-loaded Ti fiber mesh is excellent.
Vehof, J.W. *et al.* [183] (2002)	Implantation of transforming growth factor β-I-loaded titanium fiber mesh (pore size 250 μm, 86% porosity) with and without Ca-P coating into cranial defects in rabbits.	Bone ingrowth into fiber mesh was observed, however, penetration inside the mesh porosity was limited.	In the Ti-TGF-β-I close bone contact was observed and bone appeared to be denser than in Ti-CaP and Ti porous scaffold.
Kroese-Deutman, H.C. *et al.* [184] (2008)	Implantation of a Ti fiber mesh (fiber Ø 45 μm, porosity > 86%) loaded with platelet-rich plasma (PRP) into a rabbit segmental radial defect.	Bone ingrowth observed after 12 weeks. Newly formed bone was in direct contact with the Ti surface.	PRP loaded Ti scaffold exhibit a beneficial effect on bone formation.
Sargeant, T.D. *et al.* [185] (2008)	*In vitro* colonization evaluation of mouse osteoblastic cells on a Ti foam-peptide amphiphile containing phosphoserine residues and the RGDS epitope.	Bioactivity and high cell biocompatibility was accomplished in the RGDS-modified construct.	RGDS epitope concentrations used in the nanofiber networks demonstrated significant cell migration into the hybrids, proliferation and differentiation into osteoblasts.
Chen, F. *et al.* [186] (2007)	Implantation of osteoblasts precursor cells into Ti mesh-coral composite scaffold into the backs of nude mice.	Bone ingrowth observed after 2 months. New bone formed integrated well into the Ti mesh.	Ti mesh-coral composite scaffold with osteoblasts precursors cells is an efficient means to engineer segmental bone, processing the desired shape and mechanical strength.

HA: Hydroxyapatite; Si-HA: Silicon-substituted hydroxyapatite; OCP: Octacalcium phosphate; RBM: Rat bone marrow; CaP: Calcium phosphate; RGD: Arginyl-glycyl-aspartyl peptide; RGDS: Arg-gly-Asp-Ser synthetic peptide.

**Table 14:** Preclinical studies using Ta-hybrids scaffolds.

Author	Animal model & implantation site	Clinical results	Demonstrated properties
Gordon, W.J. *et al.* [187] (2005)	Ta porous scaffold cultured *in vitro* with canine and emu chondrocytes in static and dynamic environments.	Histology evaluation revealed that the tissue was heavily populated with mesenchymal cells that resembled chondrocytes. The sections cultured in dynamic bioreactors were covered with cartilaginous matrix.	Ta porous scaffold exhibited a chondroprotective function.
Bobyn, J.D. *et al.* [188] (2005)	Implantation of cylindrical porous Ta (Implex Co.), mean pore size 430 μm and 75% porosity in the intramedullary canal of the ulna of dogs accompanied with zoledronic acid intravenous dose.	Bone islands formed within the scaffold pores for both groups; however, island size was bigger in the zoledronic group.	In the zoledronic acid-treated group new bone formation was higher.
Barrère, F. *et al.* [189] (2003)	Implantation of OCP-coated and non-coated porous Ta scaffolds (mean pore size 430 μm and 75% porosity) into back muscle of goats.	After 12 weeks in the OCP coated-scaffolds bone formation in the center of the implant was observed.	OCP coating stimulated the bone ingrowth without the intervention of fibrous tissue.
Barrère, F. *et al.* [190] (2003)	Implantation of BCA-coated porous Ta scaffolds (mean pore size 430 μm and 75% porosity) into the femoral diaphysis of goats.	Bone apposition increased steadily with the implantation time in the coated scaffolds and BIC was significantly higher in the BCA-coated scaffolds (30% at 12 weeks).	BCA coating enhances the bone integration as compared to the non-coated scaffolds.
Lima, E.G. *et al.* [191] (2008)	Chondrocyte-seeded porous Ta scaffold for articular cartilage regeneration.	Osteochondral constructs developed a gradient of extracellular deposition and the developed Young’s modulus was within the range of native cartilage.	Osteochondral constructs with native cartilage properties were achieved when a Ta scaffold was employed instead of devitalized trabecular bone
Mardones, R.M. *et al.* [192] (2005)	Periosteum of rabbits was placed into porous Ta scaffolds, which were cultured under chondrogenic conditions.	Hyaline-like cartilage outgrowth was found on the surface of the scaffolds with underlying fibrous fixation.	Mechanical properties were noted to be similar to the normal rabbit cartilage.
Zou, X. *et al.* [193] (2007)	Implantation of a porous Ta ring loaded with nucleated cells in hyaluronic acid gel and rhBMP-2 in an anterior lumbar body fusion (ALIF) in pigs.	The histological appearance of the lumbar spine specimens with hyaluronic acid gel, had more mature bone in the central hole of the porous Ta ring.	Nucleated cells in hyaluronic acid gel promoted a higher bone marrow formation in the central hole of the porous Ta ring than the collagraft strips with rhBMP-2.
Sidhu, K.S. *et al.* [194] (2001)	Implantation of Ta porous scaffold (Hedrocel®) (pores averaging 500-600 μm, porosity 75-80%) modified with rhBMP-2 in the cervical spine of goats.	Bone ingrowth was observed in the rhBMP-2 modified scaffolds (12.5%) compared with (2.5%) reached by the non-modified group.	The modification with rhBMP-2 facilitated the osteoinduction within the Ta scaffold.
Li, H. *et al.* [195] (2005)	Implantation of Ta-coated carbon fibre cage loaded with Colloss® into the lumbar spine of pigs.	With clinical μCT evaluation, new bone formation could be clearly demonstrated inside the cage.	Excellent biocompatibility was demonstrated by μCT images, in which bone in direct contact with the Ta-coated cages was abundant.

OCP: Octacalcium phosphate; BCA: Bone-like carbonated apatite; BIC: Bone-implant contact; rhBMP-2: Recombinant human bone morphogenetic protein-2; COLLOSS®: Bovine extracellular matrix product containing native BMPs; μCT: micro-computer tomography.

**Table 15:** Clinical studies using titanium-ceramic, titanium-polymer, or cell loaded or autologous bone graft loaded titanium scaffolds.

Author	Animal model & implantation site	Clinical outcomes	Demonstrated properties
Thalgott, J.S. *et al.* [196] (2002)	Implantation of MOSS Ti mesh cages (DePuy Acromed, Raynham, MA) filled by coralline HA and demineralized bone matrix into 50 patients (28 to 72 years).	A solid fusion rate of 96% was achieved. Mean pain decrease was 60% overall. A total of 70% of all patients either returned to work or to home activities after ≈ 8 months after surgery.	The combination of titanium mesh cages, coralline hydroxyapatite and demineralized bone matrix is effective for anterior interbody fusion of the lumbar spine.
Thalgott, J.S. *et al.* [197] (2003)	Implantation of a cylindrical Ti mesh cages (DePuy Acromed, Raynham, MA) filled with local bone graft into 26 nonmyleopathic patients (34 to 81 years).	After 64 months 80.7% had an excellent or good clinical outcome, yielding a fusion rate of 100%. All cages remained intact with no evidence of cage settling or collapse.	Ti mesh cages filled with local bone graft and rigid anterior plating is effective for cervical reconstruction after corpectomy and a viable alternative to the use of fibular strut allograft.
Thongtrangan, I. *et al.* [198] (2003)	Implantation of a Ti vertebral body expandable cage filled with autograft, allograft and calcium phosphate into 15 patients (30 to 79 years).	Vertebral column defects could be reconstructed without significant complications after the mean follow-up time of 12.6 months.	The Ti cage provides an additional means of achieving reduction of kyphotic deformity and stabilization after tumor resection.
Niu, C.C. *et al.* [199] (2005)	Implantation of a Ti alloy cervical spinal cage (VIGOR^TM^, Central Medical Tech., Taiwan) filled with tricalcium phosphate granules (Osteograft-S, Kyocera Co., Japan) into 54 patients (35 to 66 years).	87% of the patients exhibited satisfactory clinical outcome after 3 years of follow-up. Successful fusion was obtained in 90.5 % of the operated discs.	The porous-coated Ti alloy cage provided adequate mechanical support and stability in the disc space and an excellent fusion result without subsidence of disc.
Chuang, H.C. *et al.* [200] (2006)	Implantation of Ti mesh cages (TMCs) (Mos Miami, UK) filled with autologous bone graft and triosite (calcium phosphate ceramics) into 15 patients (19 to 69 years).	11 patients experienced improvement of clinical neurological symptoms, 3 patients remained the same, and 1 patient became worse.	The clinical results of the study are acceptable. TMCs appear to provide an acceptable way to reconstruct the anterior column after corpectomy.
Boden, S.D. *et al.* [201] (2000)	Implantation of a Ti interbody fusion cages filled with rhBMP-2/collagen into 14 patients with single-level lumbar degenerative disc disease.	All patients of the rhBMP-2 group achieved true interbody fusion after 24 months, while 2 of the 3 patients treated with autogenous bone graft deemed to be fused.	The arthrodesis was found to occur more reliably in patients treated with rhBMP-2 filled fusion cages than in controls treated with autogeneous bone graft.
Regnér, L. *et al.* [202] (1998)	Implantation of a Ti fiber mesh allocated on the undersurface of a tibial prosthesis coated and un coated with HA/TCP into 36 patients undergoing total knee arthroplasty.	After 2 years, the HA/TCP tibial components displayed smaller anterior-posterior tilt and less subsidence.	HA/TCP coating on the undersurface of the tibial component improved the stability and seemed to improve the quality at the interface between the tibial component and the bone.
Hibi, H. *et al.* [203] (2006)	Implantation of one Ti mesh plate (Stryker, Kalamazoo, MI) tissue-engineered with platelet-rich plasma and autologous mesenchymal stem cells in an alveolar cleft osteoplasty of a 9-year-old female patient.	TEOM regenerated the bone in the alveolar cleft defect without donor-site morbidity resulting from the autologous bone graft.	The Ti scaffold facilitated a rigid space without disturbing the blood supply from the overlying flaps, but needed to be removed before tooth eruption.

rhBMP-2: Recombinant human bone morphogenetic protein-2; HA: Hydroxyapatite; TCP: Tricalcium phosphate; TEOM: Tissue-engineered osteogenic material.

Table 15 lists the data derived from some clinical studies using hybrid Ti constructs. Although the various approaches used in bone tissue engineering result in increased bone formation, there is a lack of long-term data able to elucidate how long this *de novo* bone formation can be maintained. Formal examination of these clinical cases is pending. Moreover, there are a number of challenges to be overcome in the transition from preclinical studies in experimental animals to clinical trials in humans. In order to allow comparisons between different preclinical studies and their outcomes, it is essential that animal models and methods to evaluate the achieved results become standardized to accomplish the accumulation of reliable data leading to the development of intelligent constructs. Furthermore, it should be kept in mind that most of the cell-loaded scaffolds studies were performed using young adult or even fetal animal cells and not with cells from elderly patients. Therefore, extensive research will be needed to determine if results can be extended to the human situation and used in a clinical situation for treating human bone defects.

## 8. Summary

Porous metallic scaffolds are used in tissue engineering to replace damaged hard tissues in order to restore its functionality. These structural scaffolds possess an imposed pore structure and interconnectivity and are designed to maintain their shape and strength through the process of repair of the injured bone. For the long-term replacement of bone defects porous metallic scaffolds offer the advantage of interfacial porosity as well as permanent structural framework. They can be made by a number of processes (e.g. powder metallurgy, decomposition of foaming agents, replication, rapid prototyping technologies, among many others). Enormous progress has been made in the development of metallic scaffolds by rapid prototyping techniques and many researchers and surgeons believe that instead of biodegradable scaffolds, biochemically-modified porous metallic scaffolds are more suitable for the development of implants for load-bearing applications. To date, there are many *in vivo* and *in vitro* tissue-culturing approaches for bone repair using metallic scaffolds with macro-porous structure. Porous metallic structures have been tested as a bone-engineered construct using the cell-based and the growth-factor-based strategies. It has been also demonstrated that coating the metallic scaffolds with various proteins such as collagen, RGD-peptide, vibronectin and fibronectin leads to accelerated osseointegration and enhanced bone formation *in vivo*. Future directions of research in this field will probably focus on the efficient combinations of osteoinductive materials, osteoinductive growth factors and cell-based tissue regeneration approach using composite constructs carriers to reconstruct and repair hard tissues. The goal is to obtain a functional replacement of the injured hard tissue in a procedure that avoids the step of bone harvesting. Therefore, a perfectly controlled hybrid scaffold still remains to be developed.
